# Metal-responsive structural transformation between artificial DNA duplexes and three-way junctions†
†Electronic supplementary information (ESI) available: Full experimental procedures, thermal denaturation experiments, ESI-MS and NMR spectra, and other experimental results. See DOI: 10.1039/c6sc00383d


**DOI:** 10.1039/c6sc00383d

**Published:** 2016-02-16

**Authors:** Yusuke Takezawa, Shuhei Yoneda, Jean-Louis H. A. Duprey, Takahiro Nakama, Mitsuhiko Shionoya

**Affiliations:** a Department of Chemistry , Graduate School of Science , The University of Tokyo , 7-3-1 Hongo , Bunkyo-ku , Tokyo 113-0033 , Japan . Email: shionoya@chem.s.u-tokyo.ac.jp

## Abstract

DNA three-way junctions (3WJs) are essential structural motifs for DNA nanoarchitectures and DNA-based materials. We report herein a metal-responsive structural transformation between DNA duplexes and 3WJs using artificial oligonucleotides modified with a 2,2′-bipyridine (bpy) ligand. A mixture of bpy-modified DNA strands and natural complementary strands were self-assembled exclusively into duplexes without any transition metal ions, while they formed 3WJs in the presence of Ni^II^ ions. This transformation was induced by the formation of an interstrand Ni^II^(bpy)_3_ complex, which served as a template for the 3WJ assembly. Altering the amount and identity of the metal ion regulated the 3WJ induction efficiency. Removal of the metal using EDTA quantitatively regenerated the duplexes. The metal-dependent structural conversion shown here has many potential applications in the development of stimuli-responsive DNA materials.

## Abstract 2

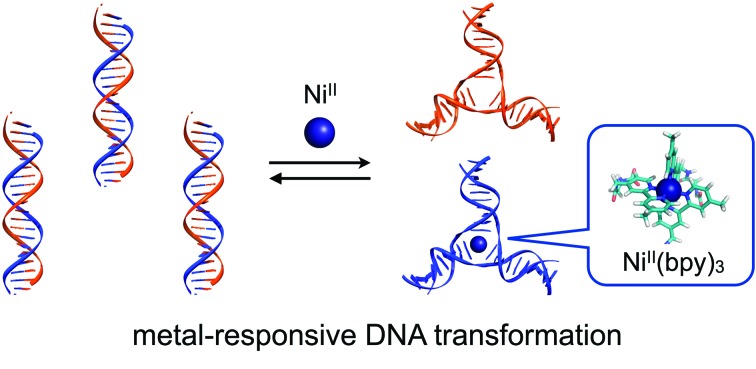
Metal-responsive structural transformation between DNA duplexes and three-way junction structures was demonstrated utilizing artificial oligonucleotides modified with a 2,2’-bipyridine ligand.

## Introduction

DNA branched structures have been proven to be an essential structural motif for developing DNA nanoarchitectures, whose structures are programmed based on the sequence-specificity of DNA hybridization.^1^ Since Seeman published a milestone paper demonstrating the construction of immobile DNA junctions,^2^ DNA branched structures, especially three-way junctions (3WJs), have been widely utilized as building blocks of two- and three-dimensional nanoscale structures^1^ as well as soft materials.^3^ In addition, 3WJs have been employed as scaffolds for molecular assembly^4^ and as reaction spaces.^5^ In light of the functional versatility of DNA 3WJ structures, it would be highly advantageous to be able to stabilize and induce these motifs.^6^ This would endow DNA-based materials with stimuli-responsiveness.

Metal–ligand coordination is one of the most exploited molecular interactions to develop stimuli-responsive supermolecules and materials.^7^ This is also the case for DNA architectures, in which a variety of interstrand metal complexes have been covalently incorporated.^8^ Notable examples include the introduction of artificial metal-mediated base pairs into DNA duplexes.^9^ This approach has yielded a wide range of metal-responsive functional DNA, whose structure,^10^ catalytic activity,^11^ as well as electrical conductivity^12^ can be regulated. In contrast, conjugation of metal complexes with other DNA structural motifs, including triplexes,^13^ quadruplexes,^14^ and junctions,^15^,^16^ has not been widely explored for the purpose so far. We have previously developed an artificial metallo-DNA 3WJ,^15^ which was composed of three oligonucleotides containing an unnatural bipyridine-modified nucleoside. Upon addition of Ni^II^ ions, the 3WJ was thermally stabilized by the formation of a tris(bipyridine) metal complex that crosslinked the three strands. A similar metallo-DNA 3WJ was thereafter exploited for constructing higher-order structures by others,^16^ suggesting its potential usefulness as a component of DNA-based materials.

**Scheme 1:**
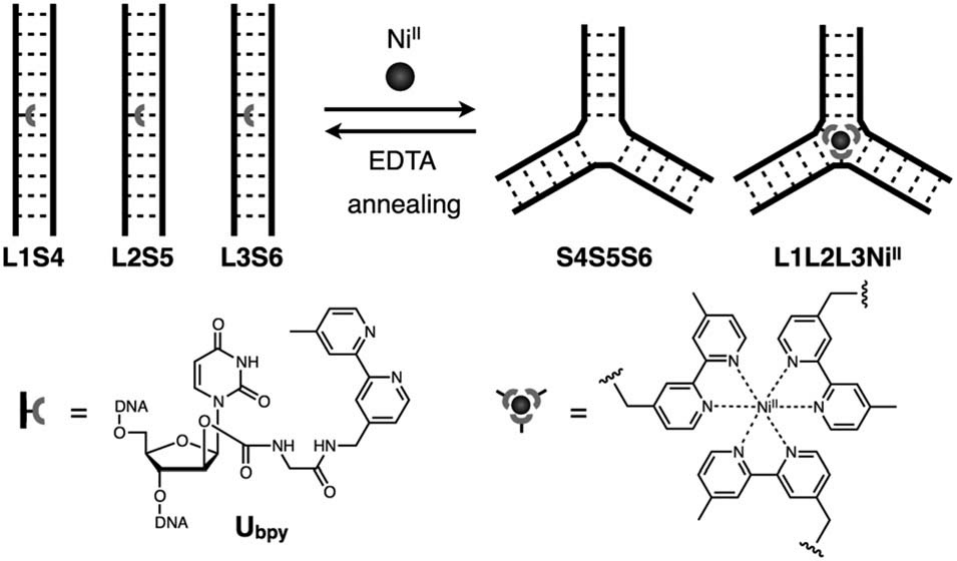
Schematic representation of the metal-responsive structural transformation between artificial DNA duplexes and three-way junctions.

In this study, we have investigated the structural transformation between DNA duplexes and 3WJ structures in response to metal coordination (Scheme 1). Such a structural reorganization involving metallo-DNA conjugates is of great use because the structural motif would be readily embedded into higher-order DNA architectures. The transformation was demonstrated with six DNA strands, whose sequences were designed so as to form both duplexes and 3WJs (Table 1). Three strands (**L1**, **L2**, and **L3**) have a bpy ligand at the middle, and the others (**S4**, **S5**, and **S6**) are complementary to the bpy-modified strands. We expected that the addition of transition metal ions would induce a structural transformation to 3WJs through the formation of a tris(bipyridine) metal complex at the junction core.

**Table 1:** Sequences of DNA strands used in this study^*a*^

DNA	Sequences (5′ to 3′)^*b*^
**L1**/**S1**	GAA GGA ACG **X**AC ACT CGC AG
**L2**/**S2**	GTT CCA CGC **X**AC GTT CCT TC
**L3**/**S3**	CTG CGA GTG **X**AG CGT GGA AC
**S4**	CTG CGA GTG TAC GTT CCT TC
**S5**	GAA GGA ACG TAG CGT GGA AC
**S6**	GTT CCA CGC TAC ACT CGC AG

^*a*^See the ESI for other strands.

^*b*^ **X** = **U_bpy_** for **L1**, **L2**, and **L3**, **X** = T for **S1**, **S2**, and **S3**.

## Results and discussion

We synthesized a novel bpy-modified nucleoside, which possesses a bpy ligand at the 2′-α position (**U_bpy_**). The novel **U_bpy_** nucleoside was efficiently incorporated into DNA strands to provide bpy-modified oligonucleotides **L1**, **L2**, and **L3** (see the ESI†). As described later, a DNA 3WJ containing three 2′-α-modified **U_bpy_** nucleotides (*i.e.***L1L2L3**) showed a higher thermal stabilization upon metal coordination compared to a 3WJ with 2′-β-modified nucleotides used in the previous study.^15^

**Fig. 1:**
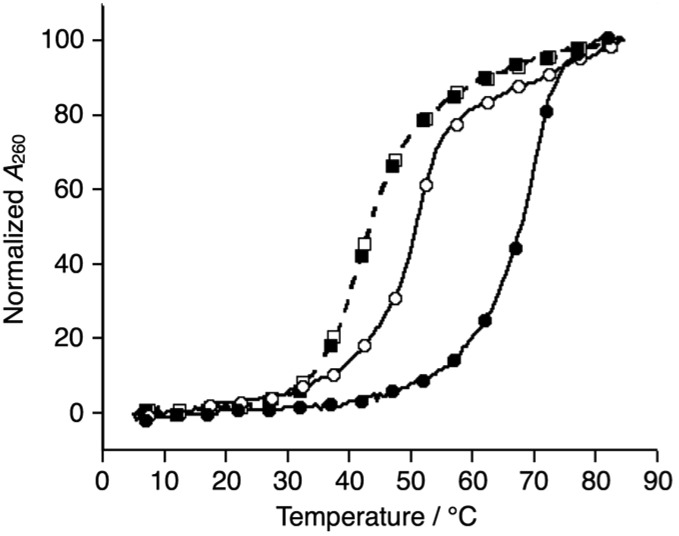
Melting curves of DNA 3WJs, **L1L2L3** (circles) and **S1S2S3** (squares), in the absence (white) and presence of Ni^II^ ions (black). [DNA strand] = 1.0 μM each, [EDTA] = 10 μM or [Ni^II^] = 1.0 μM in 10 mM MOPS buffer (pH 7.0), 100 mM NaCl, 0.2 °C min^–1^. All the samples were annealed before the measurements.

We began our investigation by determining the thermal stability of the hybridization products (Tables 2 and S3†). Bpy-modified 3WJ **L1L2L3** showed a sigmoidal melting curve with a *T*_m_ = 51.7 °C in the absence of metal ions (Fig. 1). When one equiv. of Ni^II^ ion was added, the *T*_m_ of **L1L2L3** was increased substantially to 70.5 °C (Δ*T*_m_ = +18.8 °C). Such a significant metal-dependent stabilization was not observed for other 3WJs containing fewer than three bpy ligands, *i.e.***S1S2S3** (Δ*T*_m_ = +0.5 °C), **S1S2L3** (+0.7 °C), or **S1L2L3** (+5.8 °C) (Fig. S3 and Table S2†). Thus, the stabilization was attributed to the formation of an interstrand Ni^II^(bpy)_3_ complex at the core of the 3WJ, similarly to the previously reported metallo-3WJ.^15^ The formation of the metallo-3WJ **L1L2L3·Ni^II^** was further confirmed by mass spectrometry (Fig. S4†). In contrast, *T*_m_ values of the duplexes (**L1S4**, **L2S5**, and **L3S6**), containing one bpy ligand, were not affected by the addition of Ni^II^ ions (Table S3†), precisely because of the inability to form an interstrand bipyridine–metal complex.

**Table 2:** Melting temperatures of DNA 3WJs and the DNA mixture in the absence and presence of Ni^II^ ions^*a*^

	Metal-free^*b*^	1 eq. of Ni^II^	
	*T*_m_/°C	*T*_m_/°C	Δ*T*_m_/°C
Three-way junctions			
**L1L2L3**	51.7 ± 0.3	70.5 ± 0.4	+18.8
**S1S2S3**	42.1 ± 0.3	42.6 ± 0.5	+0.5
**S4S5S6**	43.3 ± 0.5	43.0 ± 0.4	–0.3

Mixture of six strands			
**L1**, **L2**, **L3**, **S4**, **S5**, and **S6**	62.5 ± 0.3	43.7 ± 0.7	–18.8
		69.3 ± 0.6	+6.8

^*a*^Average of at least 3 runs after annealing. Standard errors are also listed.

^*b*^In the presence of 10 equiv. of EDTA.

Comparison of the melting temperatures demonstrated that the duplexes (**L1S4**, **L2S5**, and **L3S6**, *T*_m_ = 62.0, 60.3, and 65.1 °C, respectively) were more stable than the 3WJs (**L1L2L3** and **S4S5S6**, *T*_m_ = 51.7 and 43.3 °C, respectively) under metal-free conditions. This suggests that duplex formation is favored in the absence of metal ions. Conversely, addition of one equiv. of Ni^II^ ions made the bpy-modified 3WJ the most stable structure (**L1L2L3·Ni^II^**). This raised the possibility that formation of 3WJs can be induced by metal coordination.

**Fig. 2:**
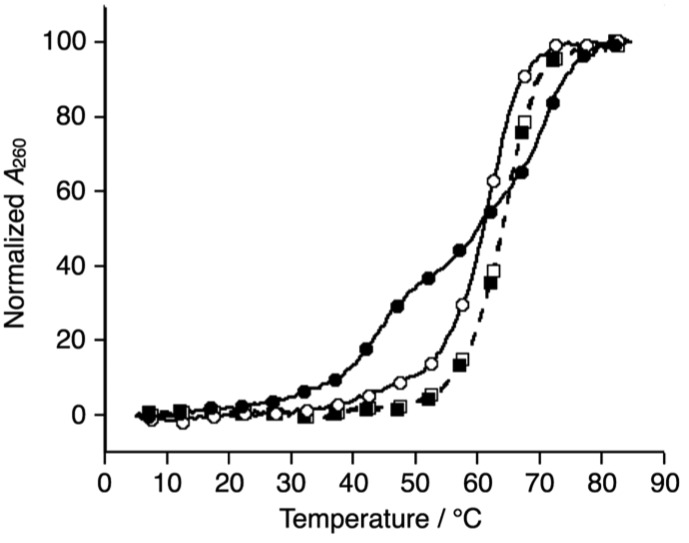
Melting curves of a mixture of the six DNA strands in the absence (white) and presence of Ni^II^ ions (black); (circles) **L1**, **L2**, **L3**, **S4**, **S5**, and **S6**; (squares) **S1**, **S2**, **S3**, **S4**, **S5**, and **S6**. The conditions were the same as for Fig. 1.

Subsequently, melting profiles of a mixture of all six strands (**L1**, **L2**, **L3**, **S4**, **S5**, and **S6**) were analyzed (Fig. 2). In the absence of metal ions, the melting curve showed a one-step transition with a *T*_m_ of 62.5 °C, which is almost an average of the *T*_m_ values of the duplexes, **L1S4**, **L2S5**, and **L3S6**. This indicated that the duplexes were predominantly formed from the six DNA strands. When one equiv. of Ni^II^ ions was added to the DNA mixture, its melting curve seemed to change to a two-step transition. The *T*_m_ value of each transition (43.7 °C and 69.3 °C) is in good agreement with those of the unmodified 3WJ **S4S5S6** (43.0 °C) and the metallo-3WJ **L1L2L3·Ni^II^** (70.5 °C), respectively. This melting profile implied preferential formation of the 3WJs (**L1L2L3·Ni^II^** and **S4S5S6**) in the presence of Ni^II^ ions.^17^ Notably, the melting curve of the mixture of six natural strands (**S1**, **S2**, **S3**, **S4**, **S5**, and **S6**) was not changed upon Ni^II^ addition, indicating that Ni^II^(bpy)_3_ complexation altered the hybridization behavior of the DNA mixture.

**Fig. 3:**
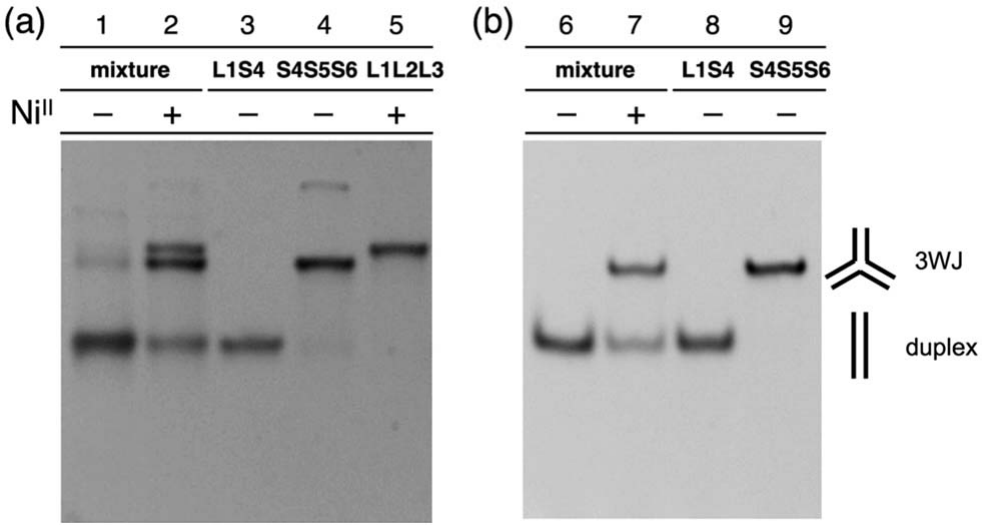
Native PAGE analysis of a mixture of **L1**, **L2**, **L3**, **S4**, **S5**, and **S6** in the absence and presence of Ni^II^ ions. (a) After SYBR Gold staining. (b) With an **S4** strand labeled with FAM. [DNA strands] = 1.0 μM each, [EDTA] = 10 μM or [Ni^II^] = 1.0 μM in 10 mM MOPS buffer (pH 7.0), 100 mM NaCl. 18% gel, TAMg buffer (pH 8), at 4 °C.

The hybridization products were then evaluated by native polyacrylamide gel electrophoresis (PAGE). Fig. 3a shows an image of the gel stained with SYBR Gold dye. The DNA mixture without metal ions showed nearly a single band corresponding to the DNA duplexes (lane 1). Upon addition of one equiv. of Ni^II^ ions, two new bands with lower mobility appeared (lane 2). These bands were ascribable to the 3WJs, **S4S5S6** and **L1L2L3·Ni^II^**, indicating that Ni^II^ addition induced the formation of the two 3WJs. To quantify the amount of the 3WJs formed, native PAGE analysis was conducted with a FAM-labeled **S4** strand (**FAM-S4**), and then the band intensities of the FAM-labeled products were compared (Fig. 3b). The results showed that the DNA duplexes were exclusively formed in the absence of the metal ions (lane 6) while 3WJs were formed in *ca.* 60% yield^17^ in the presence of Ni^II^ ions (lane 7). Such 3WJ formation was not observed when natural DNA strands were used in place of some of the bpy-modified strands (*e.g.* a mixture of **S1**, **L2**, **L3**, **S4**, **S5**, and **S6**; Fig. S7 and S8†). This reinforces the conclusion that the 3WJ formation was induced by the interstrand Ni^II^(bpy)_3_ complexation.

**Fig. 4:**
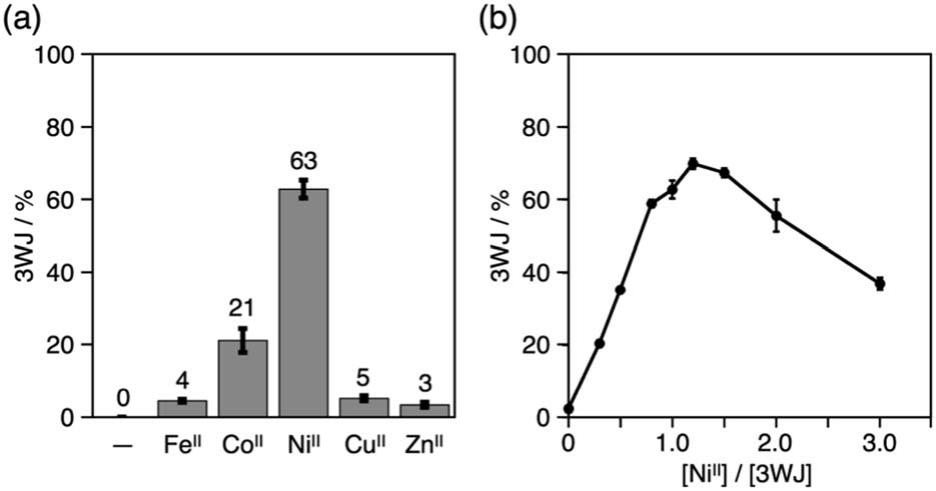
Yields of the DNA 3WJs formed in the presence of (a) various transition metal ions and (b) different amounts of Ni^II^ ions. The yields were estimated based on the native PAGE analysis. Error bars indicate the standard errors.

The yield of the 3WJs varied in response to the identity and the amount of metal ions added (Fig. S9 and S10†). The yield dropped to *ca.* 20% when Co^II^ ions were substituted for Ni^II^, while other ions showed almost no 3WJ induction effects (Fig. 4a). This presumably correlates with the large formation constant of the Ni^II^(bpy)_3_ complex (log *β*_3_ = 20.2).^15^,^18^ The efficiency of the Ni^II^-mediated 3WJ formation increased in proportion to the Ni^II^ concentration in the range of 0 to 1.2 equiv. and began to decline with excess additions (Fig. 4b). This stoichiometric behavior is almost consistent with a 3 : 1 complexation of the bpy ligand and Ni^II^ ions. These results represent a coordination-driven feature of the structural transformation using the bpy-modified DNA strands.

**Fig. 5:**
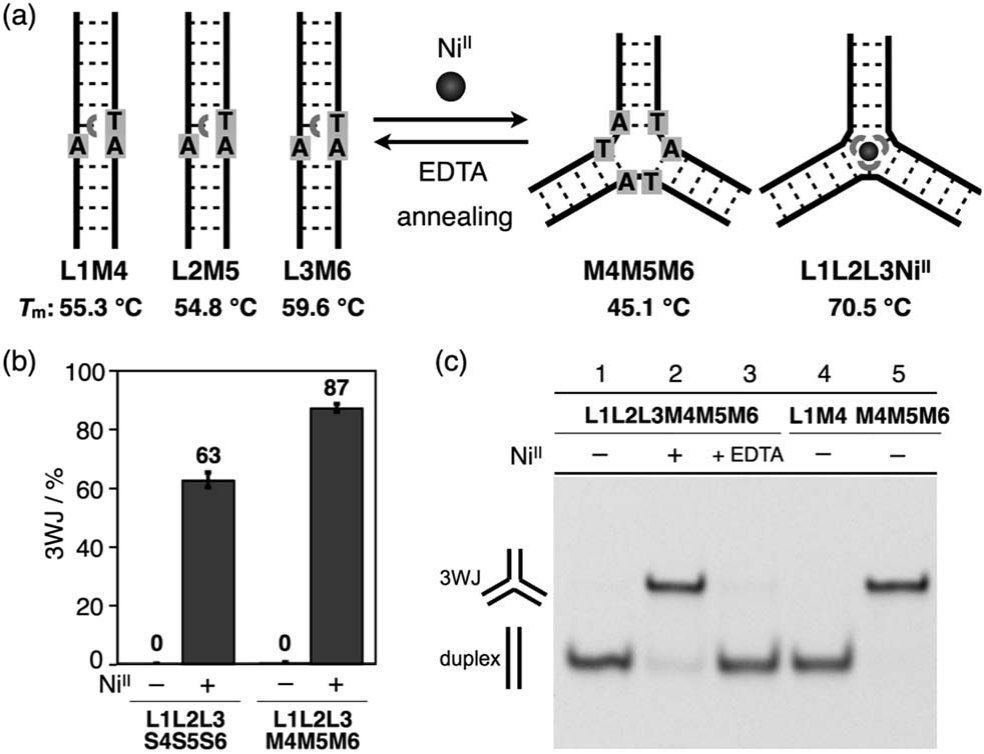
(a) Schematic representation of Ni^II^-responsive 3WJ transformation with mutated counter DNA strands (**M4**, **M5**, and **M6**). (b) Yields of the 3WJs. Error bars indicate the standard errors. (c) Native PAGE analysis of the products. **M4** was labeled with FAM.

With the goal of promoting a metal-responsive duplex-to-3WJ transformation, we redesigned the natural counter strands **M4**, **M5**, and **M6**, such that the two nucleobases in the middle were mutated from **TA** to **AT** (Fig. 5 and Table S1†). The resulting duplexes showed lower thermal stability (*T*_m_ = 55.3, 54.8, and 59.6 °C for **L1M4**, **L2M5**, and **L3M6**, respectively) than the original duplexes (**L1S4***etc.*) (Table S4†) owing to the existence of mismatch pairs (**U_bpy_**–**T** and **A**–**A**). In contrast, the 3WJ **M4M5M6** contains no mismatches and thus showed a similar thermal stability (*T*_m_ = 45.1 °C) to the original 3WJ **S4S5S6** (43.3 °C). Native PAGE analysis (Fig. 5c) revealed that alteration of the relative thermal stabilities led to more efficient 3WJ induction formation (*ca.* 90%).^19^ In addition, subsequent treatment with a chelating agent (EDTA) followed by annealing, to remove the Ni^II^ ions, led to the complete regeneration of the metal-free duplexes (lane 3). These results indicate that the Ni^II^-responsive structural transformation under these conditions is both nearly quantitative and reversible.

## Conclusions

In conclusion, we have demonstrated a metal-dependent structural transformation between DNA duplexes and 3WJs using bpy-modified artificial oligonucleotides. While the mixture of bpy-modified strands and complementary strands exclusively forms duplexes under metal-free conditions, Ni^II^ addition induces the formation of 3WJs. This transformation occurs as a result of the formation of an interstrand Ni^II^(bpy)_3_ complex. In addition, the removal of Ni^II^ ions by EDTA regenerates the duplexes, confirming the metal-ion responsiveness of the structural rearrangement. Stimuli-responsive switching of 3WJ structures has gained much attention as a versatile tool to functionalize DNA materials.^20^ Therefore, we believe that the metal-responsive 3WJ transformation presented here will provide an exciting advance in DNA nanotechnology and create new opportunities in DNA-based materials science.

## Experimental section

### Oligonucleotide synthesis

Oligodeoxynucleotides were synthesized on an Applied Biosystems 394 DNA synthesizer by standard phosphoramidite chemistry. The synthesis of the phosphoramidite derivative of **U_bpy_** is presented in the ESI.† The DNA synthesis was carried out on a 1 μmol scale in DMTr-on mode with standard reagents purchased from Glen Research. The coupling time of the nucleosides was extended to 15 min. The products were deprotected in 25% NH_3_ solution at 55 °C for 8 h. The oligomers were firstly purified and detritylated using a PolyPak II cartridge (Glen Research) and further purified by reverse-phase HPLC (Waters XBridge C18 column, 0.1 M TEAA (pH 7.0)/MeCN gradient, 60 °C) (Fig. S1†). All DNA strands were identified by MALDI-TOF mass spectrometry (see ESI†). The amount of the oligomers was determined based on the UV absorbance at 260 nm. The molar extinction coefficients (*ε*_260_) of the bpy-modified DNA strands (**L1**, **L2**, and **L3**) were estimated^15^ by the sum of the *ε*_260_ value of the bpy group and that of corresponding unmodified oligonucleotides calculated by the nearest-neighbor method. Some of the unmodified oligonucleotides purified by HPLC were purchased from Japan Bio Services and used without further purification.

### Melting analysis

All samples were prepared by mixing the DNA strands (1.0 μM) in 10 mM MOPS buffer (pH 7.0) containing 100 mM NaCl. After addition of NiSO_4_·7H_2_O (Soekawa) or EDTA, the solutions were heated to 85 °C and cooled slowly to 5 °C at the rate of 1.0 °C min^–1^. Absorbance at 260 nm was monitored by a UV-1700 spectrophotometer (Shimadzu) equipped with a TMSPC-8 temperature controller while the temperature was raised from 5 °C to 85 °C at the rate of 0.2 °C min^–1^. A drop of mineral oil was laid on the sample to prevent evaporation. Normalized absorbance shown in the figures was calculated as follows:Normalized *A*_260_ = {*A*_260_(*t* °C) – *A*_260_(5 °C)}/{*A*_260_(85 °C) – *A*_260_(5 °C)} × 100.

The melting temperature (*T*_m_) was determined as an inflection point of a melting curve using the LabSolutions *T*_m_ analysis software (Shimadzu) with a 17-point adaptive smoothing program. Average *T*_m_ values of at least 3 independent runs are shown in Tables S2–S4 in the ESI.†

### PAGE analysis

#### General procedure

Samples were prepared by mixing the DNA strands (1.0 μM) in 10 mM MOPS buffer (pH 7.0) containing 100 mM NaCl. After addition of metal sulfates (Soekawa), the solutions were heated to 85 °C and cooled slowly to 4 °C at the rate of 1.0 °C min^–1^. The gels were prepared using TAMg buffer (40 mM Tris, 76 mM MgCl_2_, 14 mM acetic acid, pH 8.0). The sample was mixed with 6× loading buffer (not containing urea or EDTA, 1 μL) and applied on an 18% gel (19 : 1). After running at 120 V for 3 h in the cool incubator (4 °C), the gels were observed using an Alpha imager mini (LMS) with a blue-LED transilluminator (Optocode). For unlabelled samples, the gels were stained with SYBR Gold (Invitrogen). Quantification of each product was accomplished by comparing the band intensities of a 3WJ **S4S5S6** (or **M4M5M6**) with that of a duplex **L1S4** (or **L1M4**), in which **S4** (or **M4**) was labeled with FAM. Averages of at least three independent experiments are shown in figures.

#### Successive transformation between duplexes and 3WJs

Six DNA strands (20 μM, 1.5 μL each) were combined in 10 mM MOPS buffer (pH 7.0) containing 100 mM NaCl to prepare the sample solutions (27 μL in total). The solutions were heated up to 85 °C and cooled slowly to 4 °C at the rate of 1.0 °C min^–1^. One third of the sample solutions (9 μL) were pipetted out and stored at –20 °C. To the residual solutions (18 μL), one equiv. of Ni^II^ ions (20 μM, 1 μL) was added. After the annealing, half of the solutions (9.5 μL) were taken out and stored at –20 °C. Subsequently, 10 equiv. of EDTA (200 μM, 0.5 μL) was added to the rest of the samples (9.5 μL), which were then annealed. All the samples were subjected to native PAGE following the general procedure outlined above (Fig. 5 and S11†).

## Supplementary Material

Supplementary information
