# Supplementary material for: Metal-responsive structural transformation between artificial DNA duplexes and three-way junctions
Source: Chem Sci. 2016 Feb 16;7(5):3006–10. doi: 10.1039/c6sc00383d (PMC6004775; doi:10.1039/c6sc00383d)
Supplement: Supplementary file 1 [file SC-007-C6SC00383D-s001.pdf]

Electronic Supplementary Material (ESI) for Chemical Science.
This journal is © The Royal Society of Chemistry 2016

Electronic Supplementary Information

Metal-responsive structural transformation between artificial DNA duplexes and three-way junctions

Yusuke Takezawa,[a] Shuhei Yoneda,[a] Jean-Louis H. A. Duprey,[a] Takahiro Nakama[a] and Mitsuhiko Shionoya[a]*

[a] Department of Chemistry, Graduate School of Science, The University of Tokyo, 7-3-1 Hongo, Bunkyo-ku, Tokyo 113-0033, Japan. E-mail: shionoya@chem.s.u-tokyo.ac.jp

Contents

1. Synthesis ·····**S2**

2. Chemical stability of the U_{bpy}-containing oligonucleotides ·····**S7**

3. Ni^{II}-dependent stabilization of the artificial three-way junction L1L2L3 ·····**S8**

4. Metal-responsive structural transformation ·····**S9**

5. Tables ·····**S12**

6. NMR spectra ·····**S14**

7. References ·····**S16**

1. Synthesis

Scheme S1. Synthesis of 2.2’-bipyridine-modified nucleoside

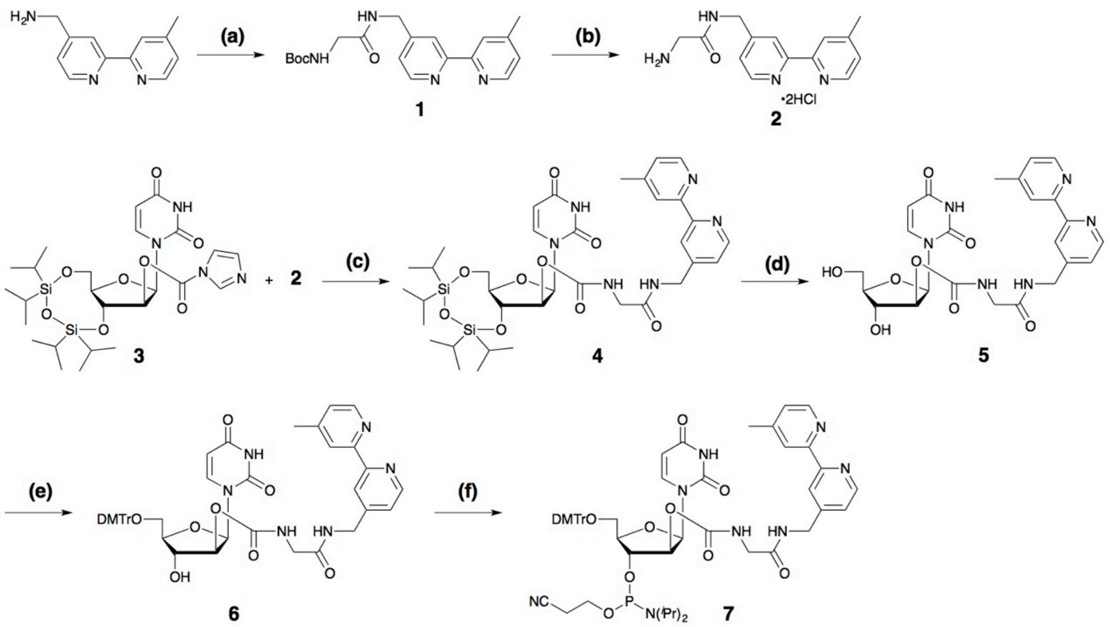

*a*Reagents and conditions: (a) *N*-Boc-glycine, EDC·HCl, CH_2Cl_2, rt, 1.5 h, 95%; (b) HCl/MeOH, rt, overnight, quant.; (c) Et_3N, CH_3CN, 70 °C, 8 h, 61%; (d) $Et_3N\cdot 3HF$, THF, rt, overnight, 90%; (e) 4,4’-dimethoxytrityl chloride (DMTrCl), pyridine, rt, 3.5 h, 91%; (f) $(^{i}Pr_2N)_2POCH_2CH_2CN$, *N*-methylimidazole, tetrazole, DMF, rt, 2 h, 76%.

General. All reactions were carried out under nitrogen atmosphere with commercial dehydrated solvents (Wako Pure Chemical Industries). The reagents for the nucleoside synthesis were purchased from Wako Pure Chemical Industries and Tokyo Chemical Industry (TCI), and were used without further purification. 4-Aminomethyl-4’-methyl-2,2’-bipyridine[1] and 2’-*O*-(imidazol-1-ylcarbonyl)-3’,5’-*O*-(tetraisopropyldisiloxane-1,3-diyl)uracil-1-β-D-arabinofuranoside[2] (**3**) were prepared according to reported procedures. Silica gel column chromatography was performed using Merck Silica Gel 60 (230–400 mesh). All NMR spectra were measured on a Bruker AVANCE 500 spectrometer (500 MHz for ^{1}H, 126 MHz for ^{13}C and 202 MHz for ^{31}P). The spectra were referenced to tetramethylsilane (TMS) in $CDCl_3$ (δ 0 ppm) or to the residual solvent signals in CD_3OD (δ 3.31 ppm) and DMSO-d_6 (δ 2.50 ppm). Electrospray ionization-time-of-flight (ESI-TOF) mass spectra were recorded on a Waters LCT Premier XE. MALDI-TOF mass spectra of DNA strands were recorded on a Bruker ultraflex spectrometer using 3-hydroxypicolinic acid (3-HPA) and diammonium citrate (DAC) as a sample matrix.

Compound 1. To a solution of 4-aminomethyl-4'-methyl-2,2'-bipyridine (124 mg, 0.62 mmol) in CH_2Cl_2 (6 mL) were added *N*-Boc-glycine (120 mg, 0.68 mmol, 1.1 eq) and 1-(3-dimethylaminopropyl)-3-ethylcarbodiimide hydrochloride (EDC·HCl, 143 mg, 0.75 mmol, 1.2 eq). The reaction mixture was stirred for 1.5 h at room temperature, diluted with CH_2Cl_2 (20 mL), and washed with water (5 mL × 3). The organic layer was dried over anhydrous Na_2SO_4, filtered, and concentrated under reduced pressure. The title compound (**1**) was obtained as a pale yellow foam (211 mg, 0.59 mmol, 95%).

^{1}H NMR (500 MHz, $CDCl_3$, 300 K): δ 8.62 (d, J = 5.0 Hz, 1H), 8.52 (d, J = 5.0 Hz, 1H), 8.28 (s, 1H), 8.23 (s, 1H), 7.23 (m, 1H), 7.15 (m, 1H), 6.59 (br, 1H), 5.13 (br, 1H), 4.58 (d, J = 6.0 Hz, 2H), 3.88 (d, J = 6.0 Hz, 2H), 2.44 (s, 3H), 1.44 (s, 9H).

^{13}C NMR (126 MHz, $CDCl_3$, 300 K): δ 169.85, 156.63, 156.23, 155.60, 149.50, 148.92, 148.28, 148.05, 124.89, 122.23, 122.13, 119.65, 80.56, 44.64, 42.42, 28.28, 21.20.

HRMS (ESI-TOF) *m*/*z*: $[M + H]^+$ calcd for $C_{19}H_{25}N_4O_3$ 357.1927, found 357.1937.

Compound 2. Compound **1** (625 mg, 1.8 mmol) was dissolved in 5–10% HCl/MeOH (TCI, 8 mL) and stirred overnight at room temperature. The reaction mixture was concentrated and dried under reduced pressure to give **2** as the HCl salt (**2**, 599 mg, 1.8 mmol, 100%) as a yellow solid.

^{1}H NMR (500 MHz, CD_3OD, 300 K): δ 8.98 (br, 1H), 8.81 (d, J = 5.2 Hz, 1H), 8.70 (d, J = 5.7 Hz, 1H), 8.66 (s, 1H), 8.45 (s, 1H) 7.87 (d, J = 5.7 Hz, 1H), 7.72 (d, J = 5.2 Hz, 1H), 4.69 (s, 2H), 3.88 (s, 2H), 2.75 (s, 3H).

^{13}C NMR (126 MHz, CD_3OD, 300 K): δ 168.02, 160.89, 154.62 149.59, 148.42, 148.31, 144.22, 129.07, 126.59, 126.12, 122.62, 43.32, 41.92, 22.42.

HRMS (ESI-TOF) *m*/*z*: $[M - H - 2Cl]^+$ calcd for $C_{14}H_{17}N_4O$ 257.1402; found: 257.1349.

Elemental analysis: Calcd for $C_{14}H_{16}N_4O \cdot 2HCl \cdot 3H_2O$: C, 43.87; H, 6.31; N, 14.62. Found: C, 44.08; H, 6.18; N, 14.29.

Compound 4. To a suspension of 2,2'-bipyridine **2** (391 mg, 1.1 mmol) and 2'-*O*-(imidazol-1-ylcarbonyl)-3',5'-*O*-(tetraisopropyldisiloxane-1,3-diyl) uracil-1-β-D-arabinofuranoside (**3**, 905 mg, 1.5 mmol, 1.3 eq) in CH_3CN (11 mL) was added triethylamine (1.3 mL, 9.4 mmol, 8 eq). The reaction mixture was heated at 70 °C for 8 h. The solvent was removed in vacuo and the residue was chromatographed on silica gel ($CHCl_3$:AcOEt:MeOH = 1:1:0–49:0:1) to afford the bipyridine-modified nucleoside **4** (535 mg, 0.70 mmol, 61%) as a pale yellow foam.

^{1}H NMR (500 MHz, $CDCl_3$, 300 K): δ 10.05 (br, 1H, exchangeable with D_2O), 8.80 (d, J = 5.1 Hz, 1H), 8.60 (d, J = 4.9 Hz, 1H), 8.40 (d, J = 1.1 Hz, 1H), 8.21 (d, J = 0.8 Hz, 1H), 7.96

(d, J = 8.2 Hz, 1H), 7.74 (dd, J = 8.2, 3.9 Hz, 1H, exchangeable with D_2O), 7.28 (dd, J = 4.9, 1.1 Hz, 1H), 7.18 (dd, J = 5.1, 0.8 Hz, 1H), 6.07 (d, J = 6.1 Hz, 1H), 5.79 (dd, J = 9.7, 6.1 Hz, 1H), 5.73 (d, J = 8.2 Hz, 1H), 5.33 (d, J = 6.1, 5.9 Hz, 1H, exchangeable with D_2O), 4.98 (dd, J = 15.6, 8.2 Hz, 1H), 4.34 (dd, J = 9.7, 9.4 Hz, 1H), 4.23–4.15 (m, 3H), 4.02 (dd, J = 13.7, 2.7 Hz, 1H), 3.88 (dd, J = 9.4, 2.3 Hz, 1H), 3.63 (dd, J = 17.6, 5.9 Hz, 1H), 2.44 (s, 3H), 1.13–0.98 (m, 28H).

^{13}C NMR (126 MHz, $CDCl_3$, 300 K): δ 168.99, 163.59, 156.43, 155.96, 155.22, 151.30, 149.58, 149.44, 148.69, 148.59, 139.63, 124.96, 122.97, 122.63, 120.12, 102.41, 82.14, 81.05, 75.91, 68.41, 59.85, 44.98, 42.34, 21.32, 17.62, 17.59, 17.44, 17.42, 17.05, 16.94, 16.83, 16.82, 13.56, 13.24, 12.97, 12.45.

HRMS (ESI-TOF) *m/z*: $[M + H]^+$ calcd for $C_{36}H_{53}N_6O_9Si_2$: 769.3412; found: 769.3427.

Compound 5. To a solution of nucleoside **4** (181 mg, 0.24 mmol) in THF (0.6 mL) was added triethylamine trihydrofluoride (0.10 mL, 0.62 mmol, 2.5 eq). The reaction mixture was stirred for 3 h at room temperature, and then THF (1 mL) was added. After further stirring overnight, $CHCl_3$ (6 mL) was added to precipitate the product. The precipitate was collected and washed with $CHCl_3$ (6 mL × 2). The resulting solid was coevaporated with pyridine (20 mL) and CH_3CN (15 mL × 2) to remove the residual reagent. The target compound **5** was obtained (113 mg, 0.21 mmol, 90%) as a reddish solid. While a ^{1}H NMR spectrum at 333 K showed a single species, a spectrum at 300 K showed two sets of signals, indicating the existence of two conformers.

^{1}H NMR (500 MHz, DMSO-d_6, 333 K): δ 11.09 (br, 1H), 8.58 (s, 1H), 8.53 (s, 1H), 8.30 (br, 1H), 8.27 (s, 1H), 8.21 (s, 1H), 7.64 (d, J = 8.0 Hz, 1H), 7.40 (br, 1H), 7.28 (m, 2H), 6.14 (d, J = 4.8 Hz, 1H), 5.60 (br, 1H), 5.55 (d, J = 8.0 Hz, 1H), 5.04 (dd, J = 4.0, 4.0 Hz, 1H), 4.84 (br, 1H), 4.39 (d, J = 5.3 Hz, 2H), 4.10 (s, 1H), 3.80 (dd, J = 9.8, 4.8 Hz, 1H), 3.69-3.57 (m, 4H), 2.42 (s, 3H).

^{1}H NMR (500 MHz, DMSO-d_6, 300 K): *major conformer*, δ 11.31 (d, J = 2.0 Hz, 1H), 8.60 (d, J = 4.9 Hz, 1H), 8.55 (d, J = 4.9 Hz, 1H), 8.46 (t, J = 6.0 Hz, 1H), 8.29 (s, 1H), 8.24 (s, 1H), 7.68 (d, J = 8.1 Hz, 1H), 7.64 (t, J = 6.0 Hz, 1H), 7.30 (m, 2H), 6.14 (d, J = 4.8 Hz, 1H), 5.75 (d, J = 5.0 Hz, 1H), 5.58 (dd, J = 8.1, 2.0 Hz, 1H), 5.06-4.99 (m, 2H), 4.41 (d, J = 6.0 Hz, 2H), 4.10 (dd, J = 9.1, 4.8 Hz, 1H), 3.80 (dd, J = 9.7, 4.8 Hz, 1H), 3.70-3.55 (m, 4H), 2.42 (s, 3H).

^{13}C NMR (126 MHz, DMSO-d_6, 300 K): *major conformer*, δ 169.02, 163.02, 155.26, 155.00, 154.93, 150.19, 149.50, 149.12, 148.97, 147.91, 141.24, 124.94, 122.40, 121.26, 118.94, 100.84, 83.91, 82.77, 77.06, 72.56, 60.06, 43.51, 41.42, 20.70.

HRMS (ESI-TOF) *m/z*: $[M + Na]^+$ calcd for $C_{24}H_{26}N_6O_8Na$ 549.1710, found 549.1698.

Compound 6. Nucleoside **5** (214 mg, 0.41 mmol) was coevaporated with pyridine (4 mL × 2) and dissolved in pyridine (4 mL), to which 4,4'-dimethoxytrityl chloride (182 mg, 0.54 mmol, 1.3 eq) was added. The reaction mixture was stirred for 3.5 h at room temperature, subsequently diluted with $CHCl_3$ (150 mL), and washed with H_2O (60 mL). The aqueous layer was extracted with $CHCl_3$ (60 mL × 2). The organic extracts were then combined and dried over anhydrous Na_2SO_4. The solvent was removed under reduced pressure and the residual oil was subjected to silica gel column chromatography ($CHCl_3$:MeOH = 1:0–1:1 containing 0.5% triethylamine). The target molecule **6** was obtained (306 mg, 0.37 mmol, 91%) as a white solid.

^1H NMR (500 MHz, DMSO-d_6, 333 K): δ 11.19 (br, 1H), 8.58 (d, *J* = 4.7 Hz, 1H), 8.53 (d, *J* = 4.9 Hz, 1H), 8.34 (t, *J* = 5.4 Hz, 1H), 8.27 (s, 1H), 8.22 (s, 1H), 7.50 (d, *J* = 8.1 Hz, 1H), 7.40-7.39 (m, 2H), 7.34-7.20 (m, 9H), 6.90-6.89 (m, 4H), 6.15 (d, *J* = 5.2 Hz, 1H), 5.71 (br, 1H), 5.36 (d, *J* = 8.1 Hz, 1H), 5.06 (s, 1H), 4.41 (m, 2H), 4.14 (m, 1H), 3.89 (m, 1H), 3.74 (s, 6H), 3.71–3.57 (m, 2H), 3.35–3.30 (m, 2H), 2.42 (s, 3H).

^1H NMR (500 MHz, DMSO-d_6, 300 K): *major conformer*, δ 11.31 (br, 1H), 8.59 (d, *J* = 4.9 Hz, 1H), 8.53 (d, *J* = 5.0 Hz, 1H), 8.44 (t, J = 6.0 Hz, 1H), 8.28 (s, 1H), 8.23 (s, 1H), 7.53 (d, *J* = 8.1 Hz, 1H), 7.40–7.38 (m, 2H), 7.34–7.24 (m, 9H), 6.91–6.89 (m, 4H), 6.14 (d, *J* = 5.3 Hz, 1H), 5.81 (d, *J* = 5.1 Hz, 1H), 5.34 (d, *J* = 8.1 Hz, 1H), 5.07 (dd, *J* = 5.3, 5.2 Hz, 1H), 4.40 (m, 2H), 4.14–4.13 (m, 1H), 3.89–3.87 (m, 1H), 3.73 (s, 6H), 3.69–3.43 (m, 4H), 2.41 (s, 3H).

^{13}C NMR (126 MHz, DMSO-d_6, 300 K): *major conformer*, *δ*169.01, 162.95, 158.13, 155.28, 155.02, 154.98, 150.16, 149.50, 149.14, 148.98, 147.92, 144.74, 135.45, 135.29, 129.77, 127.88, 127.72, 126.78, 124.95, 122.42, 121.28, 118.95, 113.24, 100.99, 85.72, 81.23 (two signals should be overlapped), 77.24, 72.77, 62.55, 55.04, 43.52, 41.44, 20.71.

HRMS (ESI-TOF) *m/z*: $[M + Na]^+$ calcd for $C_{45}H_{44}N_6O_{10}Na$ 851.3016, found 851.3000.

Compound 7. To a solution of nucleoside **6** (300 mg, 0.37 mmol), tetrazole (21 mg, 0.31 mmol, 0.8 eq) and *N*-methylimidazole (6 μL, 0.08 mmol, 0.2 eq) in DMF (1.9 mL) was added 2-cyanoethyl-*N,N,N',N'*-tetraisopropylphosphorodiamidite (180 μL, 0.57 mmol, 1.5 eq). The reaction mixture was stirred for 2 h at room temperature. The reaction mixture was diluted with AcOEt (80 mL) and washed with water (80 mL × 2). The organic layer was dried over anhydrous Na_2SO_4 and then evaporated to dryness. The residue was purified by silica gel column chromatography ($CHCl_3$:AcOEt = 1:1 then $CHCl_3$:MeOH = 9:1 containing 0.5%

triethylamine) to afford compound **7** (288 mg, 0.28 mmol, 76%, diastereomeric mixture) as a pale yellow foam. The obtained phosphoramidite **7** was coevaporated with dry MeCN and immediately used for DNA synthesis. ^{1}H NMR and ^{31}P NMR spectra indicated the existence of two diastereomers.

^{1}H NMR (500 MHz, $CDCl_3$, 300 K): δ 8.74-8.70 (d × 2, *J* = 5.0 Hz, 1H), 8.63 (d, *J* = 5.0 Hz, 1H), 8.40 (d, *J* = 7.0 Hz, 1H), 8.24 (s, 1H), 8.22–8.03 (d × 2, *J* = 8.0 Hz, 1H), 7.44–7.41 (m, 2H), 7.37–7.29 (m, 7H), 7.20–7.17 (m, 1H), 6.90–6.86 (m, 4H), 6.23–6.19 (d × 2, *J* = 6.0 Hz, 1H), 5.76–5.65 (dd × 2, *J* = 6.0, 6.0 Hz, 1H), 5.57–5.51 (m, 1H), 5.29–5.22 (d × 2, *J* = 8.0 Hz, 4H), 4.90–4.80 (m, 2H), 4.69–4.64 (m, 1H), 4.38–4.30 (m, 2H), 4.11–4.01 (m, 2H), 3.83–3.82 (d × 2, *J* = 1.0 Hz, 6H), 3.72–3.49 (m, 4H), 2.59 (t, *J* = 6.0 Hz, 1H), 2.46 (s, 3H), 2.41 (t, *J* = 6.0 Hz, 1H), 1.28–1.04 (m, 12H).

^{31}P NMR (202 MHz, $CDCl_3$, 300 K): δ 150.98, 149.75.

HRMS (ESI-TOF) *m/z*: $[M + Na]^+$ calcd for $C_{54}H_{61}N_8O_{11}PNa$ 1051.4095, found 1051.4122.

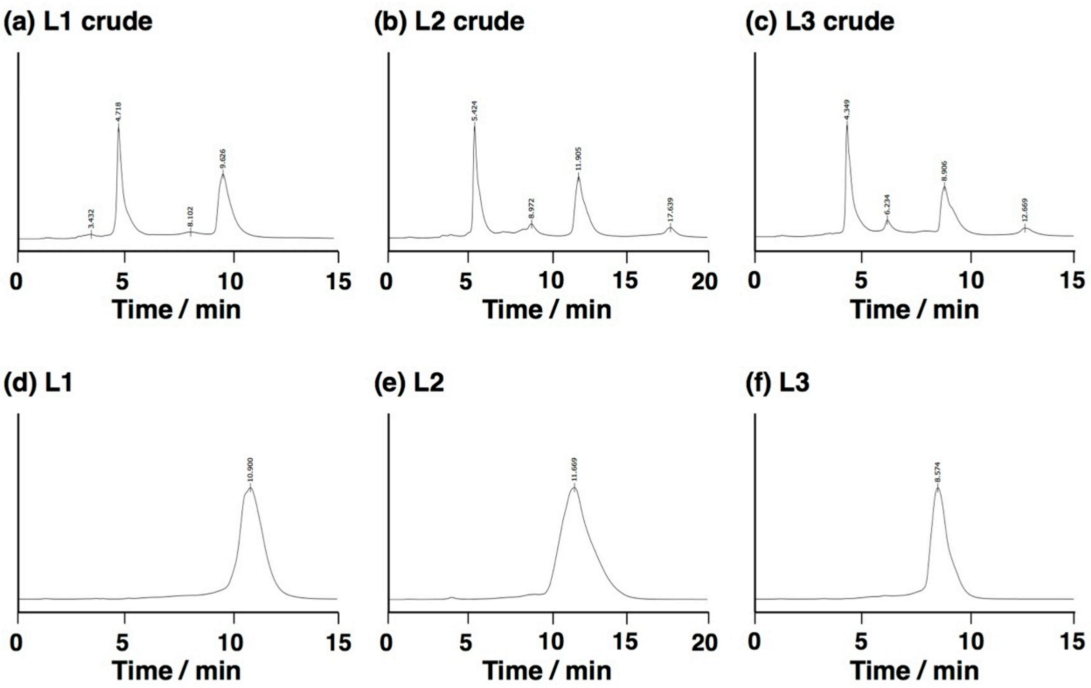

Figure S1. HPLC analysis of the bpy-modified DNA strands before (a-c) and after (d-f) purification. Waters XBridge C18 column (4.6 × 50 mm), flow rate: 1.0 mL/min, temperature: 60 °C, gradient: 0 to 20 min, 7% A to 9 % A (solvent A = MeCN, solvent B = 0.1 M TEAA buffer (pH 7.0) + 2% MeCN). Monitored at 260 nm. The second largest peaks were identified as the desired product (**L1**, **L2**, and **L3**) whereas the largest peaks were assigned to DNA strands whose bpy-moieties were cleaved during the deprotection step (for example, (c) retention time: 4.3 min**,** ESI-TOF MS *m/z* calcd for $[C_{195}H_{243}N_{80}O_{119}P_{19} - 7H]^{7-}$: 884.57; found: 884.58).

Characterization of the DNA stands possessing U_{bpy} nucleosides.

L1. HPLC retention time: 9.6 min. MALDI-TOF MS *m/z* calcd for $[C_{209}H_{255}N_{88}O_{116}P_{19} - H]^-$: 6443.3; found: 6442.6. $\varepsilon_{260} = 2.11 \times 10^5$.

L2. HPLC retention time: 11.9 min. MALDI-TOF MS *m/z* calcd for $[C_{206}H_{260}N_{67}O_{126}P_{19} - H]^-$: 6278.2; found: 6279.6. $\varepsilon_{260} = 1.81 \times 10^5$.

L3. HPLC retention time: 8.9 min. MALDI-TOF MS *m/z* calcd for $[C_{210}H_{257}N_{84}O_{121}P_{19} - H]^-$: 6481.3; found: 6482.8. $\varepsilon_{260} = 2.02 \times 10^5$.

2. Chemical stability of the U_{bpy}-containing oligonucleotides

Prior to the metal complexation experiments, the chemical stability of DNA strands containing $\mathbf{U_{bpy}}$ bases was assessed by HPLC analysis. A trinucleotide, 5′-T$\mathbf{U_{bpy}}$T-3′, was prepared and treated with 1.0 equiv. of $NiSO_4$ in the reaction buffer, (10 mM MOPS, 100 mM NaCl), under three different conditions: (a) incubation at 20 °C for 20 h, (b) annealing from 85 °C to 4 °C (1 °C/min), and (c) incubation at 85 °C for 6 h. After the addition of EDTA, the samples were analyzed by HPLC (Figure S2). Although ~6% of the carbamate linker was cleaved after incubation at 85 °C to give a cleaved nucleoside **U′**, the cleavage was not observed after the annealing process. Thus, it can be concluded that $\mathbf{U_{bpy}}$ moiety would be intact under the condition for the following experiments.

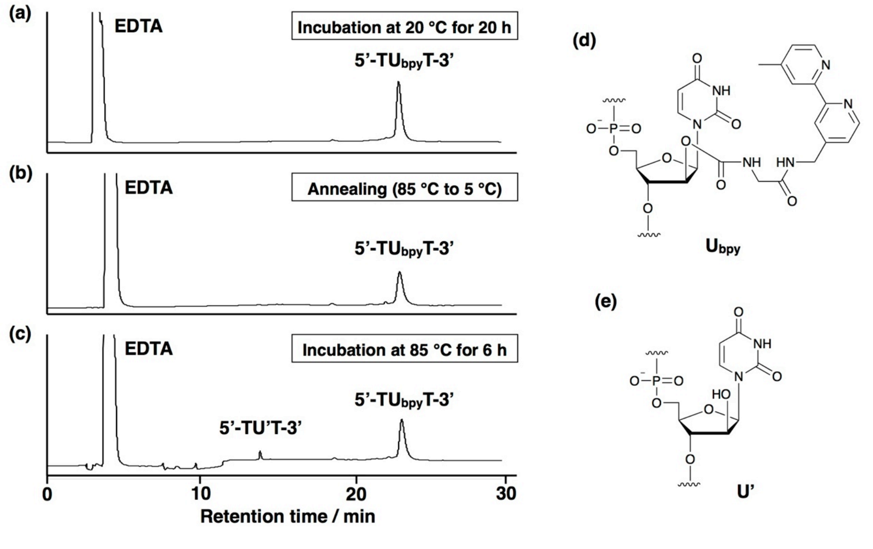

Figure S2. HPLC analysis of the bpy-modified DNA strands, 5′-T$\mathbf{U_{bpy}}$T-3′, after the treatment with Ni^{II} ions. (a) Incubated at 20 °C for 20 h, (b) annealed from 85 °C to 5 °C (1 °C/min), and (c) incubated at 85 °C for 6 h. TOSOH TSK gel ODS-80Ts (4.6 × 250 mm), flow rate: 1.0 mL/min, temperature: 60 °C, gradient: 0 to 30 min, 0% A to 30% A (solvent A = MeCN, solvent B = 0.1 M TEAA buffer (pH 7.0) + 2% MeCN). (d, e) Chemical structures of $\mathbf{U_{bpy}}$ and a cleaved nucleoside **U′**.

3. Ni^{II}-dependent stabilization of the artificial three-way junction L1L2L3

Mass spectra. Electrospray ionization-time-of-flight (ESI-TOF) mass spectra were recorded on a Waters Micromass LCT premier. The samples were prepared in 20 mM NH_4OAc buffer (pH 8.0) and annealed just before the measurements. The conditions were as follows: negative mode; capillary voltage, 2000 V; sample cone voltage, 20 V; aperture 1 voltage 40 V; desolvation temperature, 120 °C; source temperature, 20 °C.

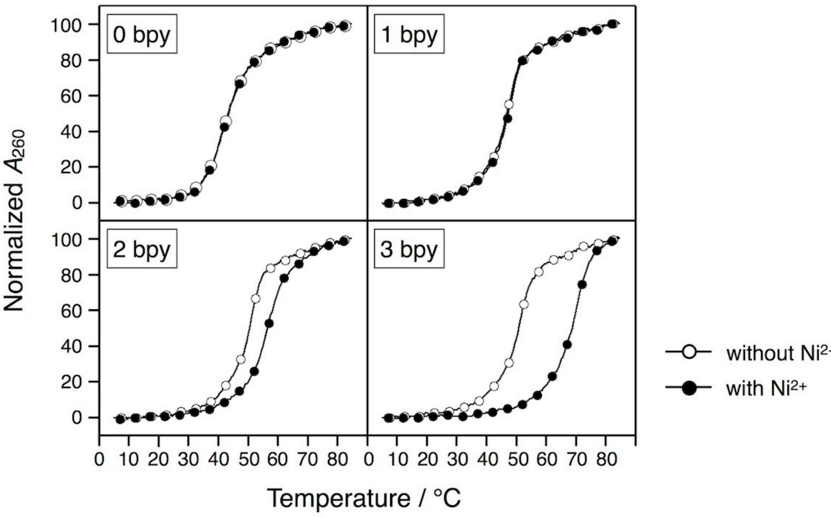

Figure S3. Melting curves of the DNA three-way junctions modified with different numbers of bipyridine units. 0 bpy: **S1S2S3**, 1 bpy: **S1S2L3**, 2 bpy: **S1L2L3**, 3 bpy: **L1L2L3**. (○) In the presence of 10 equiv. of EDTA, (●) in the presence of 1.0 equiv. of Ni^{II} ions. [DNA three-way junction] = 1.0 µM, [EDTA] = 10 µM or [Ni^{II}] = 1.0 µM in 10 mM MOPS buffer (pH 7.0), 100 mM NaCl, 0.2 °C/min.

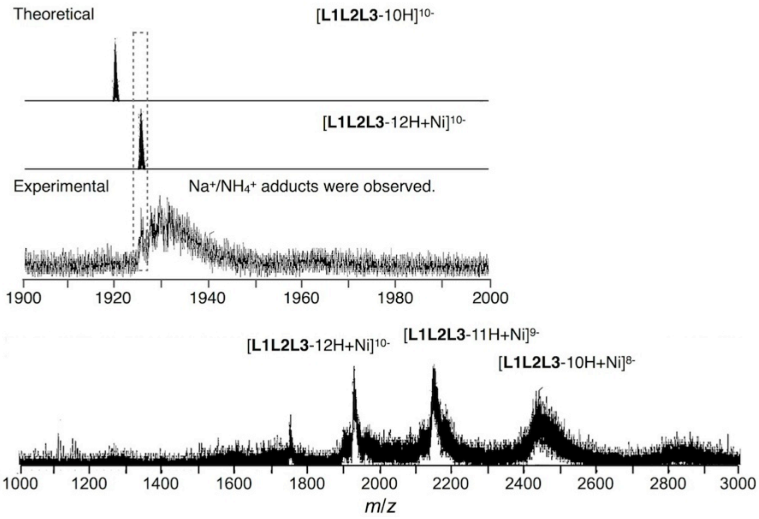

Figure S4. An ESI-MS spectrum of the metallo-DNA three-way junction **L1L2L3**·Ni^{II}. [**L1L2L3**·Ni^{II}] = 20 µM in 20 mM NH_4OAc buffer (pH 8.0). **L1L2L3** in the figure represents a neutral three-way

junction structure ($C_{625}H_{772}N_{239}O_{363}P_{57}$). The metal-free three-way junction (**L1L2L3**) was not observed under these conditions, while **L1L2L3**·Ni^{II} was observed with adducts of Na^+ or NH_4^+ ions.

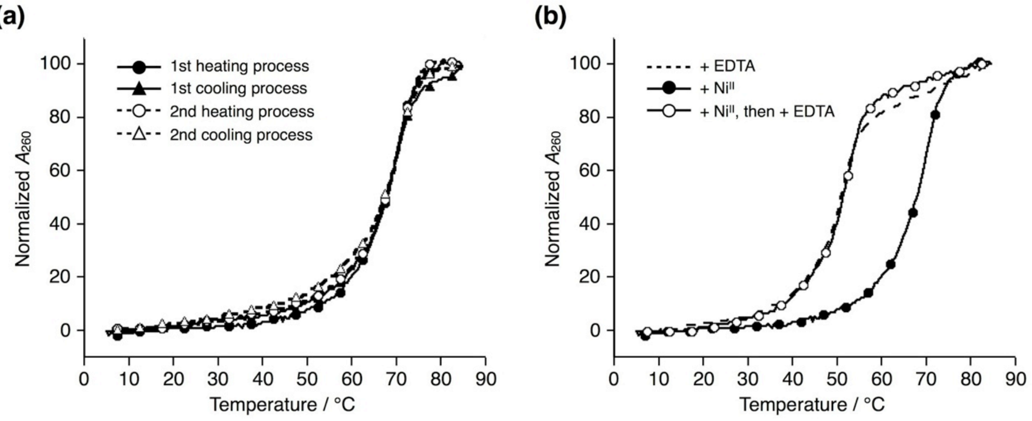

Figure S5. (a) Melting curves of **L1L2L3** in the presence of Ni^{II} ions during repeated heating-cooling cycles. After the sample annealing, melting curves were recorded during heating (●), cooling (▴), subsequent heating (○), and cooling processes (▵). **(b)** Melting curves of **L1L2L3** after successive addition of Ni^{II} ions and EDTA. (---) **L1L2L3** + EDTA, (●) **L1L2L3** + Ni^{II}, (○) **L1L2L3** + Ni^{II} was annealed and then EDTA was added. [**L1L2L3**] = 1.0 µM, [EDTA] = 10 µM or [Ni^{II}] = 1.0 µM in 10 mM MOPS buffer (pH 7.0), 100 mM NaCl, 0.2 °C/min

4. Metal-responsive structural transformation

Melting analysis. The melting analysis of a mixture of six DNA strands (**L1**, **L2**, **L3**, **S4**, **S5** and **S6** etc.) was performed according to the procedure described in the main text. The simulated melting curve of a mixture of duplexes was calculated as follows:

Abs(duplexes) = Abs(**L1S4**) + Abs(**L2S4**) + Abs(**L3S6**),

where Abs(**L1S4**), Abs(**L2S5**), and Abs(**L3S6**) are the absorbances measured in the presence of 10 equiv. of EDTA for DNA duplexes **L1S4**, **L2S5**, and **L3S6**, respectively. The simulated melting curve of a mixture of 3WJs was calculated as follows:

Abs(3WJs) = Abs(**L1L2L3·Ni^{II}**) + Abs(**S4S5S6**),

where Abs(**L1L2L3·Ni^{II}**) and Abs(**S4S5S6**) are the absorbances of 3WJ **L1L2L3** measured in the presence of 1.0 equiv. of Ni^{II} ions and that of **S4S5S6** in the presence of 10 equiv. of EDTA, respectively. The simulated melting curve shown in Figure S6 was calculated as follows:

$$\text{Abs} = \alpha\cdot\text{Abs(3WJ)} + (1 - \alpha)\cdot\text{Abs(duplex)}\ ,$$

where α represents the proportion of the 3WJs.

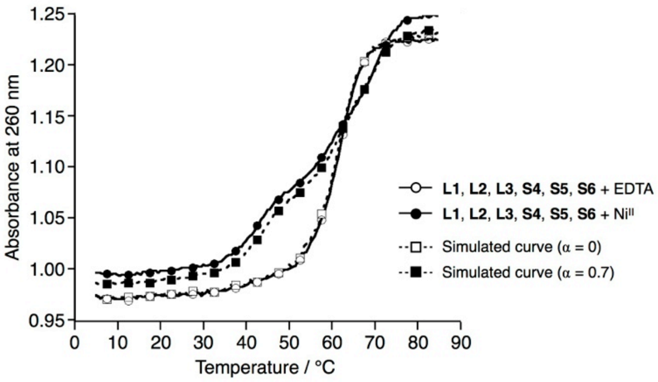

Figure S6. Melting curves of a mixture of six strands **L1**, **L2**, **L3**, **S4**, **S5**, and **S6** in the absence and presence of Ni^{II} ions. (○) Mixture + EDTA, (●) mixture + 1.0 equiv. of Ni^{II} ions. (□) A melting curve simulated by the summation of those of three metal-free duplexes **L1S4**, **L2S5**, and **L3S6** (i.e. $\alpha = 0$), (■) a melting curve simulated on an assumption that the 3WJs were formed in 70% yield (i.e. $\alpha = 0.7$). [DNA] = 1.0 μM each, [EDTA] = 10 μM or [Ni^{II}] = 1.0 μM in 10 mM MOPS buffer (pH 7.0), 100 mM NaCl, 0.2 °C/min.

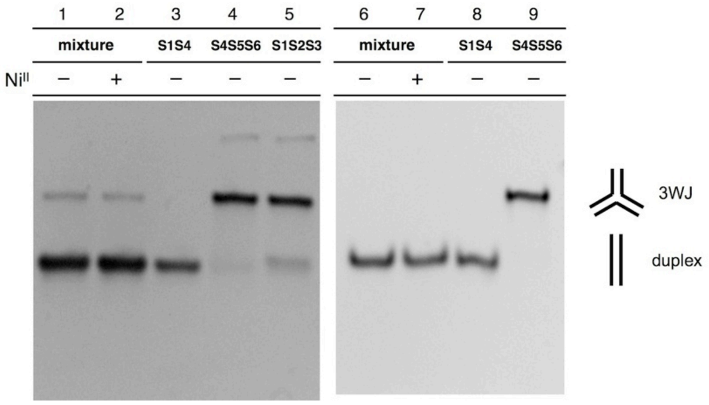

Figure S7. Native PAGE analysis of a mixture of DNA strands, **S1**, **S2**, **S3**, **S4**, **S5**, and **S6**. (Lanes 1–5) The bands were detected after a SYBR gold stain, (lanes 6–9) **S4** strand was labeled with FAM, and the bands were detected by FAM fluorescence. (Lanes 1, 6) a mixture of six strands in the presence of 10 equiv. EDTA, (lanes 2, 7) a mixture of six strands in the presence of 1.0 equiv. of Ni^{II} ions, (lanes 3, 8) duplex **S1S4**, (lanes 4, 9) three-way junction **S4S5S6**, (lane 5) three-way junction **S1S2S3**. [DNA strands] = 1.0 μM each, [EDTA] = 10 μM or [Ni^{II}] = 1.0 μM in 10 mM MOPS buffer (pH 7.0), 100 mM NaCl.

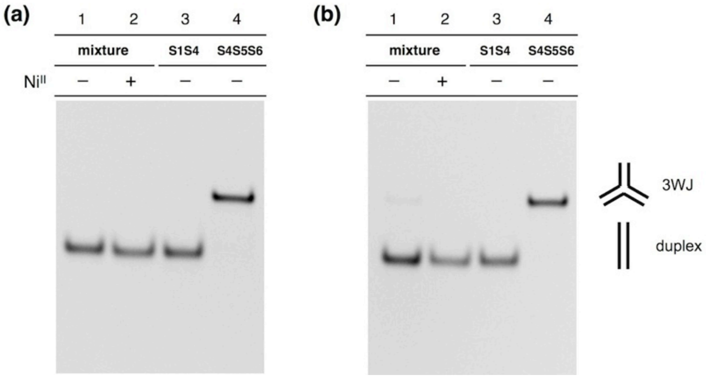

Figure S8. Native PAGE analysis of a mixture of DNA strands. **(a) S1**, **S2**, **L3**, **S4**, **S5**, and **S6**, **(b) S1**, **L2**, **L3**, **S4**, **S5**, and **S6**. **S4** strand was labeled with FAM. (Lane 1) a mixture of six strands in the presence of 10 equiv. of EDTA, (lane 2) a mixture of six strands in the presence of 1.0 equiv. of Ni^{II} ions, (lane 3) duplex **S1S4**, and (lane 4) three-way junction **S4S5S6**. [DNA strands] = 1.0 μM each, [EDTA] = 10 μM or [Ni^{II}] = 1.0 μM in 10 mM MOPS buffer (pH 7.0), 100 mM NaCl. The bands were detected by FAM fluorescence.

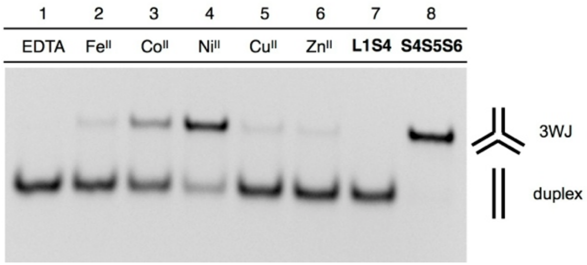

Figure S9. Native PAGE analysis of a mixture of DNA strands, **L1**, **L2**, **L3**, **S4**, **S5**, and **S6**, in the presence of various transition metal ions. **S4** strand was labeled with FAM. (Lane 1) a mixture of six strands in the presence of 10 equiv. of EDTA, (lanes 2–6) a mixture of six strands in the presence of 1.0 equiv. of Fe^{II}, Co^{II}, Ni^{II}, Cu^{II}, or Zn^{II} ions, respectively, (lane 7) duplex **S1S4**, (lane 8) three-way junction **S4S5S6**. [DNA strands] = 1.0 μM each, [EDTA] = 10 μM or [metal ions] = 1.0 μM in 10 mM MOPS buffer (pH 7.0), 100 mM NaCl. The bands were detected by FAM fluorescence.

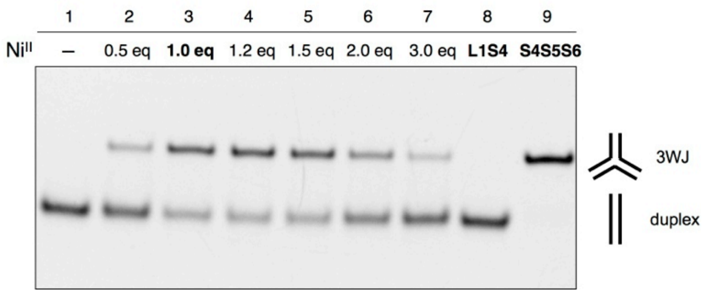

Figure S10. Native PAGE analysis of a mixture of DNA strands, **L1**, **L2**, **L3**, **S4**, **S5**, and **S6**, in the presence of different amount of Ni^{II} ions. **S4** strand was labeled with FAM. (Lane 1–7) In the presence of 0, 0.5, 1.0, 1.2, 1.5, 2.0, and 3.0 equiv. of Ni^{II} ions, respectively, (lane 8) duplex **S1S4**, and (lane 9) three-way junction **S4S5S6**. [DNA strands] = 1.0 μM each, [metal ions] = 0–3.0 μM in 10 mM MOPS buffer (pH 7.0), 100 mM NaCl. The bands were detected by FAM fluorescence.

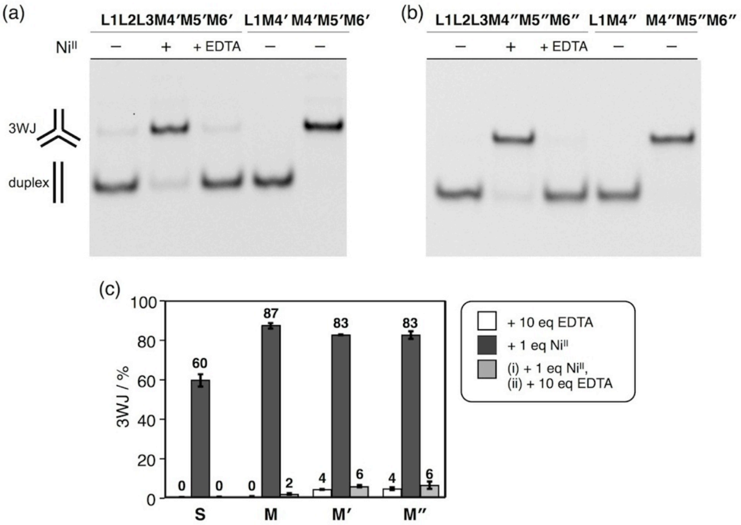

Figure S11. Native PAGE analysis of mixtures of the bpy-modified DNA strands (**L1**, **L2**, and **L3**) and mutated counter strands. For their sequences, see Table S1. **(a)** A mixture of **L1**, **L2**, **L3**, **M4′**, **M5′**, and **M6′** (with **GC** in the middle). **(b)** A mixture of **L1**, **L2**, **L3**, **M4″**, **M5″**, and **M6″** (with **CG** in the middle). **(c)** Yields of the induced 3WJs. [DNA strands] = 1.0 μM each, [EDTA] = 10 μM or [Ni^{II}] = 1.0 μM in 10 mM MOPS buffer (pH 7.0), 100 mM NaCl. The bands were detected by FAM fluorescence.

5. Tables

Table S1. Sequences of DNA strands used for the experiments shown in Figs. 5 and S11.

Strands	Sequences
**M4**	5'-CTG CGA GTG **AT**C GTT CCT TC-3'
**M5**	5'-GAA GGA ACG **AT**G CGT GGA AC-3'
**M6**	5'-GTT CCA CGC **AT**C ACT CGC AG-3'
**M4′**	5'-CTG CGA GTG **GC**C GTT CCT TC-3'
**M5′**	5'-GAA GGA ACG **GC**G CGT GGA AC-3'
**M6′**	5'-GTT CCA CGC **GC**C ACT CGC AG-3'
**M4″**	5'-CTG CGA GTG **CG**C GTT CCT TC-3'
**M5″**	5'-GAA GGA ACG **CG**G CGT GGA AC-3'
**M6″**	5'-GTT CCA CGC **CG**C ACT CGC AG-3'

Table S2. Melting temperatures of DNA three-way junctions containing zero-to-three bpy ligands in the absence and presence of one equiv. of Ni^{II} ions[a]

	Metal-free[b]	1 eq of Ni^{II}	
	T_m / °C	T_m / °C	ΔT_m / °C[c]
**S1S2S3 (0 bpy)**	42.1 ± 0.3	42.6 ± 0.5	+0.5 ± 0.6
**S1S2L3 (1 bpy)**	47.7 ± 0.1	48.4 ± 0.5	+0.7 ± 0.5
**S1L2L3 (2 bpy)**	51.8 ± 0.7	57.6 ± 0.4	+5.8 ± 0.8
**L1L2L3 (3 bpy)**	51.7 ± 0.3	70.5 ± 0.4	+18.8 ± 0.5

[a] Average of at least 3 runs after annealing (1 °C/min). Standard errors are also listed. [b] In the presence of 10 equiv. of EDTA. [c] ΔT_m represents the difference in the T_m values relative to that of the metal-free DNAs.

Table S3. Melting temperatures of DNA duplexes, three-way junctions and a mixture of six strands (**L1**, **L2**, **L3**, **S4**, **S5**, and **S6**) in the absence and presence of one equiv. of Ni^{II} ions[a]

	Metal-free[b]	1 eq of Ni^{II}	
	T_m / °C	T_m / °C	ΔT_m / °C[c]
**Three-way junctions**			
**L1L2L3**	51.7 ± 0.3	70.5 ± 0.4	+18.8 ± 0.5
**S1S2S3**	42.1 ± 0.3	42.6 ± 0.5	+0.5 ± 0.6
**S4S5S6**	43.3 ± 0.5	43.0 ± 0.4	–0.3 ± 0.6
**Duplexes**			
**L1S4**	62.0 ± 0.4	61.4 ± 0.3	–0.6 ± 0.5
**L2S5**	60.3 ± 0.5	60.6 ± 0.0	+0.3 ± 0.5
**L3S6**	65.1 ± 0.6	64.4 ± 0.6	–0.7 ± 0.8
**Mixture of 6 strands**			
**L1**, **L2**, **L3**, **S4**, **S5** and **S6**	62.5 ± 0.3	43.7 ± 0.7	–18.8 ± 0.8
		69.3 ± 0.6	+6.8 ± 0.7

[a] Average of at least 3 runs after annealing (1 °C/min). Standard errors are also listed. [b] In the presence of 10 equiv. of EDTA. [c] ΔT_m represents the difference in the T_m valuse relative to that of the metal-free DNAs.

Table S4. Melting temperatures of DNA duplexes, three-way junctions and a mixture of six strands, **L1**, **L2**, **L3**, **M4**, **M5**, and **M6** in the absence and presence of one equiv. of Ni^{II} ions[a]

	Metal-free[b]	1 eq of Ni^{II}	
	T_m / °C	T_m / °C	ΔT_m / °C[c]
**Three-way junctions**			
**L1L2L3**	51.7 ± 0.3	70.5 ± 0.4	+18.8
**M4M5M6**	45.1 ± 0.8	44.9 ± 0.7	–0.2
**Duplexes**			
**L1M4**	55.3 ± 0.3	56.0 ± 0.2	+0.7
**L2M5**	54.8 ± 0.6	55.5 ± 0.6	+0.7
**L3M6**	59.6 ± 0.1	59.9 ± 0.4	+0.3
**Mixture of 6 strands**			
**L1**, **L2**, **L3**, **M4**, **M5**, and **M6**	56.7 ± 0.4	46.8 ± 0.5	–9.9
		71.3 ± 1.0	+14.6

[a] Average of at least 3 runs after annealing (1 °C/min). Standard errors are also listed. [b] In the presence of 10 equiv. of EDTA. [c] ΔT_m represents the difference in the T_m values relative to that of the metal-free DNAs.

6. NMR spectra

Compound 1

^1H NMR (500 MHz, 300 K, $CDCl_3$)

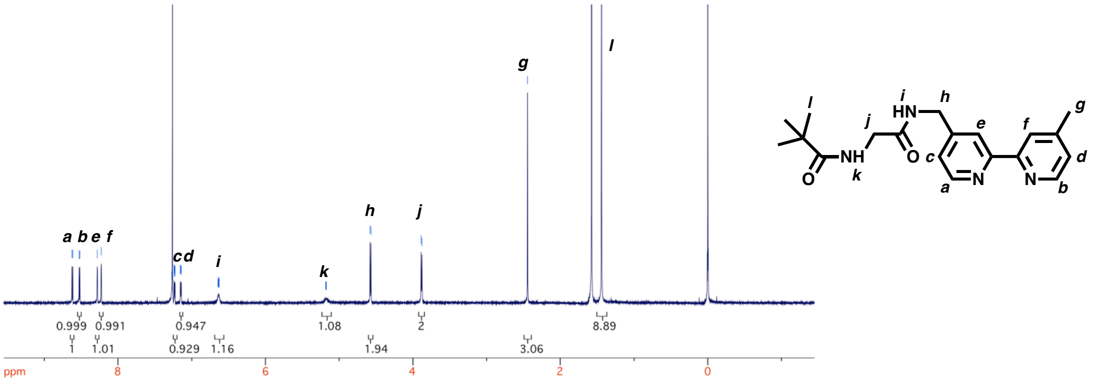

^{13}C NMR (126 MHz, 300 K, $CDCl_3$)

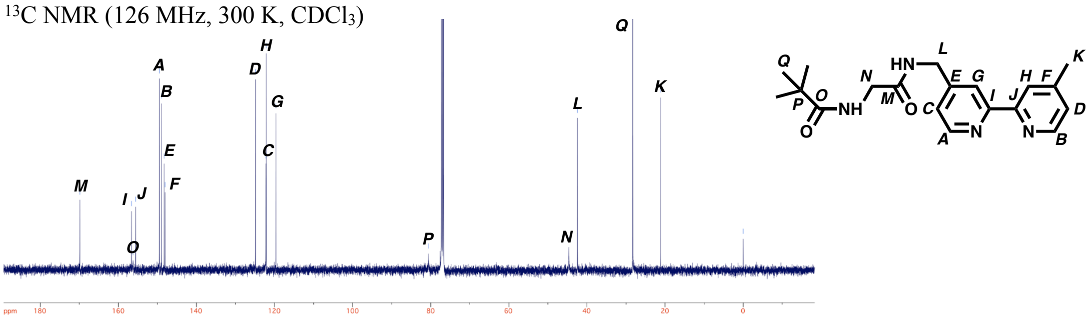

Compound 2

^1H NMR (500 MHz, 300 K, CD_3OD)

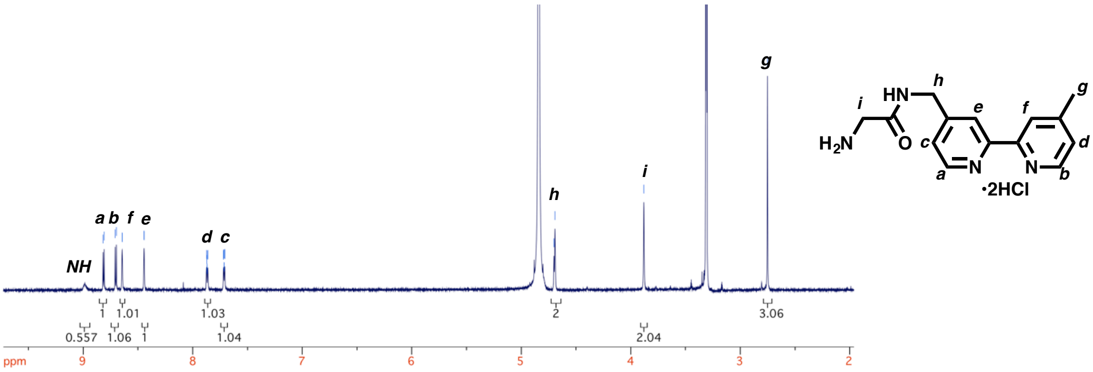

^{13}C NMR (126 MHz, 300 K, CD_3OD)

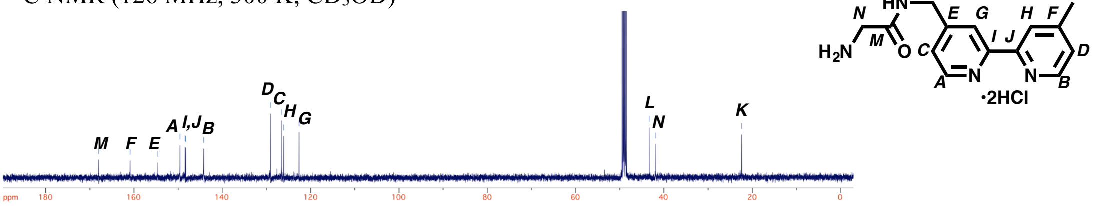

Compound 4

^{1}H NMR (500 MHz, 300 K, $CDCl_3$)

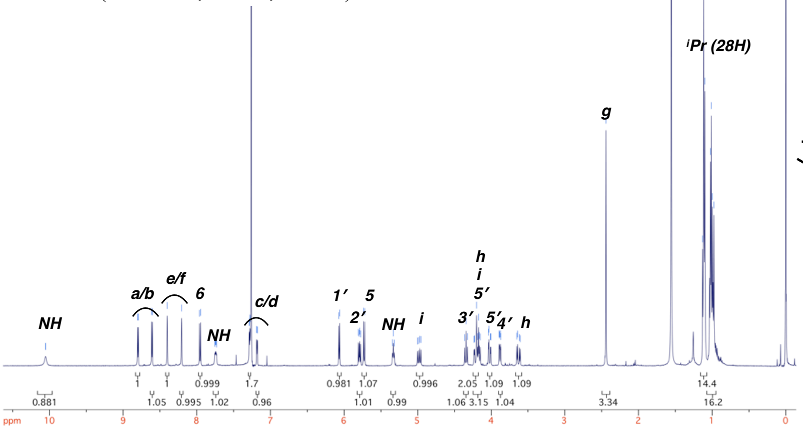

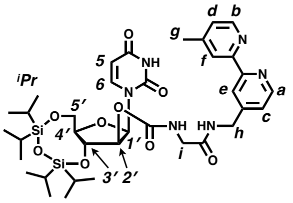

^{13}C NMR (126 MHz, 300 K, $CDCl_3$)

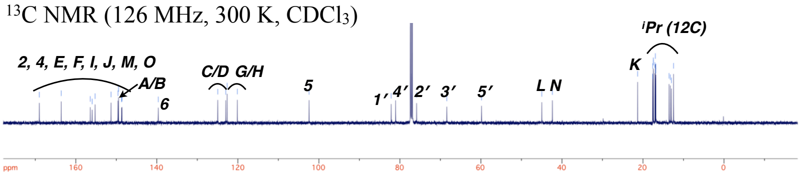

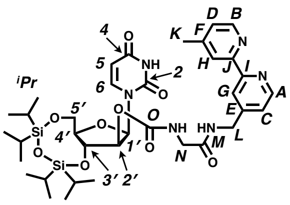

Compound 5

^{1}H NMR (500 MHz, 300 K, DMSO-d_6)

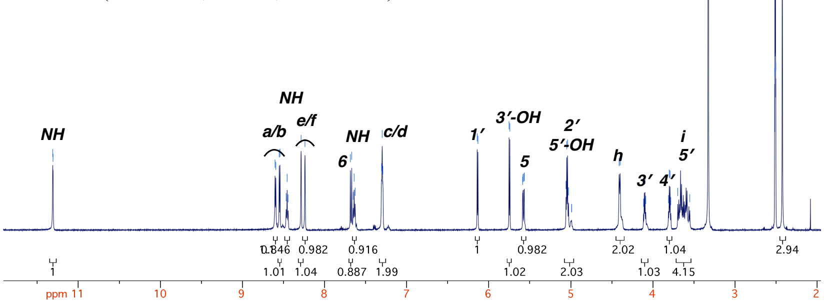

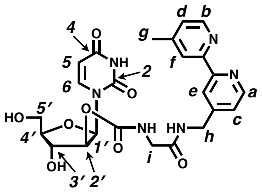

^{13}C NMR (126 MHz, 300 K, DMSO-d_6)

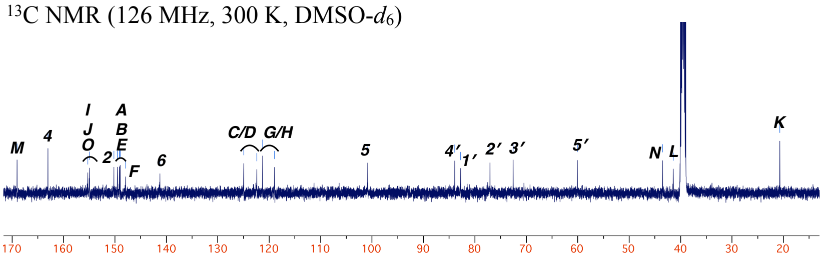

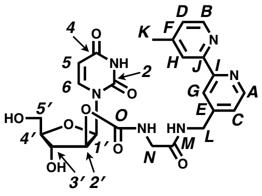

Compound 6

^{1}H NMR (500 MHz, 300 K, DMSO-d_6)

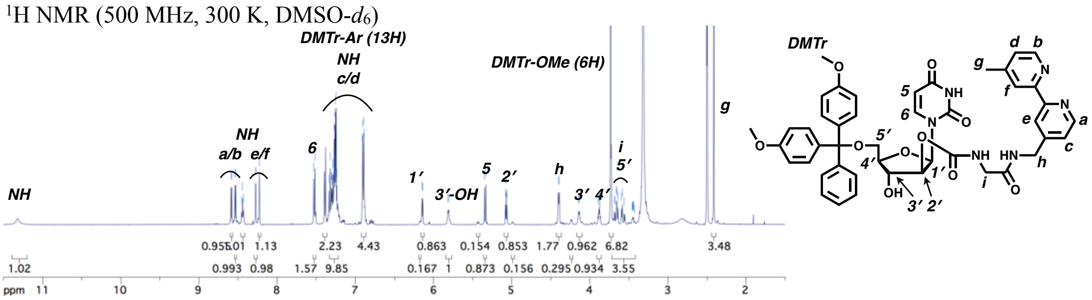

^{13}C NMR (126 MHz, 300 K, DMSO-d_6)

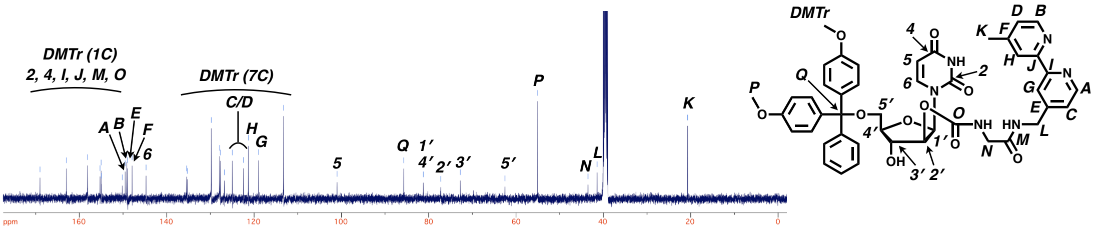

Compound 7

^{1}H NMR (500 MHz, 300 K, $CDCl_3$)

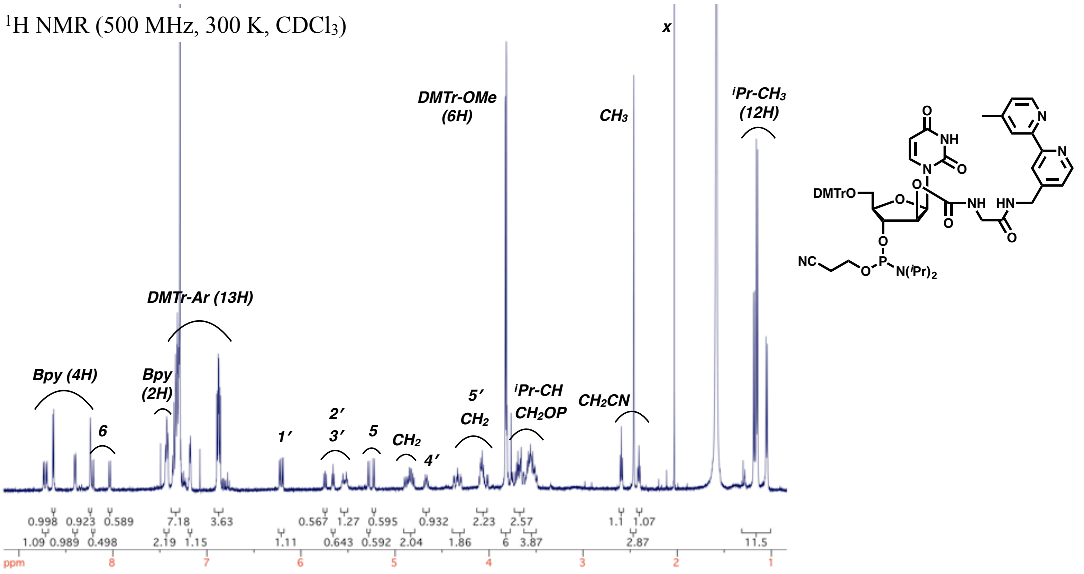

^{31}P NMR (202 MHz, 300 K, $CDCl_3$)

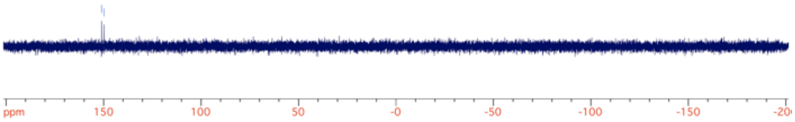

7. References

[1] N. Mano, V. Soukharev, A. Heller, *J. Phys. Chem. B* **2006**, *110*, 11180–11187.

[2] N. N. Dioubankova, A. D. Malakhov, D. A. Stetsenko, M. J. Gait, P. E. Volynsky, R. G. Efremov, V. A. Korshun, *ChemBioChem* **2003**, *4*, 841–847.
